# Changes to the Bacterial Microbiome in the Rhizosphere and Root Endosphere of *Persea americana* (Avocado) Treated With Organic Mulch and a Silicate-Based Mulch or Phosphite, and Infested With *Phytophthora cinnamomi*

**DOI:** 10.3389/fmicb.2022.870900

**Published:** 2022-04-28

**Authors:** Qurrat Ul Ain Farooq, Giles Edward St. John Hardy, Jen A. McComb, Peter Campbell Thomson, Treena Isobel Burgess

**Affiliations:** ^1^Phytophthora Science and Management, Harry Butler Institute, Murdoch University, Perth, WA, Australia; ^2^Institute of Agricultural Sciences, University of the Punjab, Lahore, Pakistan; ^3^ArborCarbon, ROTA Compound Murdoch University, Murdoch, WA, Australia; ^4^Sydney School of Veterinary Science, The University of Sydney, Camden, NSW, Australia

**Keywords:** soil amendments, silicate-based mulch, microbial profile, rhizosphere soil, phosphite

## Abstract

Plant growth and responses of the microbial profile of the rhizosphere soil and root endosphere were investigated for avocado plants infested or not infested with *Phytophthora cinnamomi* and the changes were compared in plants grown with various soil additives or by spraying plants with phosphite. Soil treatments were organic mulches or silica-based mineral mulch. Reduction of root growth and visible root damage was least in the infested plants treated with phosphite or mineral mulch applied to the soil. Rhizosphere soils and root endospheres were analyzed for bacterial communities using metabarcoding. Bacterial abundance and diversity were reduced in infested rhizospheres and root endospheres. The presence or absence of mineral mulch resulted in greater diversity and larger differences in rhizosphere community composition between infested and non-infested pots than any other treatment. Some rhizosphere bacterial groups, especially Actinobacteria and Proteobacteria, had significantly higher relative abundance in the presence of *Phytophthora*. The bacterial communities of root endospheres were lower in abundance than rhizosphere communities and not affected by soil treatments or phosphite but increased in abundance after infection with *P. cinnamomi*. These findings suggested that the addition of silicate-based mineral mulch protects against *Phytophthora* root rot, which may be partly mediated through changes in rhizosphere bacterial community composition. However, the changes to the microbiome induced by spraying plants with phosphite are different from those resulting from the application of mineral mulch to the soil.

## Introduction

Most soil-borne disease-causing agents are acclimatized to live in bulk soil and cause plant diseases when changes in physical and biotic soil conditions allow them to colonize the rhizosphere and damage plants (Avis et al., 2008). Changes in cultural practices or the application of soil additives resulting in improved disease suppression is thought to function by altering the soil microbiota. However, it is not usually known which soil microbes are impacted, and which changes lead to the desired outcome (Bakker et al., 2015). As high throughput sequencing techniques allow the analysis of the composition of the entire soil microbial population, they will provide information on how microbial populations change in the presence of a pathogen, and in response to soil additives will provide a better understanding of which microbes enhance or reduce disease expression. The molecular techniques mean that this analysis is no longer restricted to microbes easy to isolate and culture. In the longer term, this knowledge may allow more efficient exploitation of living microorganisms to suppress disease using eco-friendly, non-chemical methods.

Avocadoes are cultivated in at least 59 countries in tropical and subtropical regions. *Phytophthora* root rot or avocado wilt complex caused by the oomycete *Phytophthora cinnamomi* Rands (Ramirez-Gil et al., 2017, 2018; Hardham and Blackman, 2018) is one of the worst diseases for avocados worldwide, especially in Australia. *Phytophthora* root rot can cause losses from 45 to 90% in avocado (Molano, 2007; Perez-Jimenez, 2008) and may increase to 100% if appropriate controls are not adopted. Management of *Phytophthora* root rot is usually achieved by using chemical sprays such as phosphite and metalaxyl (Pegg et al., 1987; Dobrowolski et al., 2008; Ramirez-Gil et al., 2017; Belisle et al., 2019), but most growers adopt an integrated management strategy. This includes site management to reduce waterlogging and minimize disease spread by personnel or machinery, as well as the application of inorganic fertilizers, such as calcium, phosphorus, magnesium, and silicon, which are shown to reduce the impact of the disease (Dann and Le, 2017; Ramirez-Gil et al., 2017). The addition of organic matter as mulches, manures, and composts is a major part of an integrated strategy (Bulluck and Ristaino, 2002; Bonanomi et al., 2007; Klein et al., 2011; Ando et al., 2014; Gilardi et al., 2016; van Bruggen et al., 2016).

Organic soil additives add nutrients and improve water holding capacity (Bhadha et al., 2017; Oldfield et al., 2018). They may also reduce plant disease either directly by reducing the population of pathogenic bacteria and fungi in the soil, or indirectly by increasing the abundance of bacteria that induce systemic disease resistance in the host plant (Zhang et al., 1998; Hoitink and Boehm, 1999; Aviles et al., 2011). For example, compost enhances the density of soil bacteria that have antibiotic activity against *Fusarium oxysporum, F. solani*, and *Rhizoctonia solani* (Jambhulkar et al., 2015) through enhancement of populations of strains of *Pseudomonas, Streptomyces*, or *Bacillus* spp. Of particular interest here are reports of some Proteobacteria or Actinobacteria suppressing *Phytophthora* root rot in avocado (You et al., 1996; Yin et al., 2004; Cazorla et al., 2007; Guevara-Avendano et al., 2018). In some cases, commercial preparations of beneficial bacteria have been effective for disease control in crops such as solanaceous vegetables, fruit crops, other vegetables, and ornamentals (Junaid et al., 2013; Hu et al., 2016; Mukta et al., 2017), but in a pot trial of avocado, probiotics containing *Bacillus* spp. did not reduce root damage from *P. cinnamomi* (Farooq et al., 2022).

Junaid et al. (2013) reviewed direct and indirect mechanisms by which soil microbes might suppress a pathogen. The direct mechanisms include hyperparasitism, nutrient competition (Pal and Gardener, 2006; Jambhulkar et al., 2015), commensalism (Yoon et al., 1977; Chisholm et al., 2006), mutualism (Bronstein, 1994; Chisholm et al., 2006), and the production of antibiotic compounds (Garbeva et al., 2006, 2011; Postma et al., 2008). Indirect mechanisms include stimulation of plant growth through the production of plant growth-promoting hormones and siderophores (Bhattacharjee and Dey, 2014), and disease protection through the induction of systemic host resistance (Nakkeeran et al., 2004; Bent, 2006).

Mineral soil conditioners, particularly silicate-based ones, have shown encouraging outcomes for plant disease control and crop growth (Pozza et al., 2015; Tubana et al., 2016). Silica (Si) is not an essential element for plant growth but has been categorized as “quasi-essential.” A recent review by Rajput et al. (2021) shows there is a wide array of positive effects of silica on plants and soil microbiota, and that application of silicate-based nanoparticles may be more efficacious than conventional application. Conventional silica application has been effective for the control of anthracnose (*Colletotrichum lindemuthianum;* Moraes et al., 2009) and powdery mildew (*Sphaerotheca fuliginea*) (Menzies et al., 1992; Belanger et al., 2003). Although most of the research has been on herbaceous crops, Dann and Le (2017) showed a silica soil amendment improved root biomass, enhanced new root growth, and reduced root necrosis in avocado seedlings infected with *P. cinnamomi* or *Calonectria ilicicola*. It appears silica has its effect both through changes to the plant metabolism and the soil microbiome. Silica accumulation in the plant results in a physical barrier in the cell wall (Samuels et al., 1991; Fawe et al., 2001) as well as the stimulation of plant defense enzymes such as lipoxygenase polyphenol oxidase, peroxidase, and phenylalanine ammonia lyase (Fauteux et al., 2005; Shetty et al., 2011; Prabhu et al., 2012). It alters soil microbial diversity and richness in rhizosphere soil, but its impact varies with the season (Gao et al., 2022). It may promote beneficial bacteria (Li et al., 2019), but one study showed it could also decrease populations of beneficial bacteria such as *Rhizobacteria*. However, the application of silicate-based nanoparticles did not have this detrimental effect (Rajput et al., 2021).

Treatment of plants with pesticides can have an impact on the soil microbiome, which can be beneficial or detrimental (Lo, 2010). Phosphite is widely used to control *P. cinnamomi* damage in avocado orchards, and there is little data on how this might affect the soil microbiome. In tomato crops, phosphite has a beneficial effect; it suppresses *Ralstonia solanacearum*, and, when applied in conjunction with the biocontrol agent *Bacillus amyloliquefaciens, it* enhances its antagonistic activity (Su et al., 2021b).

In non-infested avocado plants, the addition of organic mulch, a silicate-based mineral mulch, and organic mulch, or spraying plants with phosphite increased total root growth and fine root growth (Farooq et al., 2022) (Supplementary Table S1). When *P. cinnamomi* was present, mineral mulch or phosphite treatments increased fine root weight and reduced root damage (Farooq et al., 2022). The silicate-based mineral mulch resulted in almost the same level of protection against *P. cinnamomi* as phosphite (Farooq et al., 2022). The current study focuses on the changes in microbial consortia in root endospheres and rhizospheres of avocado infested with *P. cinnamomi*, and how these changes are modulated by the application of organic mulches (chicken manure, jarrah (*Eucalyptus marginata*) wood mulch, and avocado mulch), a silicate-based mineral mulch in addition to the organic ones, or spraying the plants with phosphite. The rhizosphere and root microbial populations were analyzed using amplicon 16S primers.

Firstly, we hypothesized that the abundance and diversity of bacteria in the microbiome would increase in response to the addition of organic mulch, and there would be further changes with the addition of mineral mulch or spraying plants with phosphite. Secondly, the presence of *P. cinnamomi* would change the microbial profiles of the soils in similar ways, regardless of the soil additives. Thirdly, the abundance and diversity of the bacteria in the microbiome would be greatest in the treatments that suppressed *Phytophthora* root damage. Fourthly, since avocado root damage from *phytophthora* is reduced to a similar extent by the silicate-based mineral mulch or by spraying plants with phosphite, these two treatments will induce similar changes in the bacteria in the rhizosphere and root endosphere.

## Materials and Methods

### Glasshouse Experiment

Rhizosphere soil and avocado root tips were collected from plants grown in polybags in the glasshouse under conditions that simulated the integrated control measures for *P. cinnamomi* used by many avocado growers. Details are given in Farooq et al. (2022). Briefly, 5-month-old plants were transplanted into 150 × 380 mm (7 L) free-draining polybags (Garden City Plastics, Forrestfield, Western Australia) containing a well-drained clay loam soil with good porosity and water holding capacity from an avocado growing area in Carabooda, Western Australia and mixed 1:1 with river sand. There were 20 plants in each treatment, and 38 days after transplanting, the soil of ten of these were inoculated with *P. cinnamomi* (isolate MP 94-48 from the *Phytophthora* Science and Management culture collection, Genbank Accession number for ITS gene region is JX113294). Plants were harvested 12 weeks after inoculation and root damage and growth parameters were assessed (Farooq et al., 2022). These data showed that applying organic mulches, one of the two silicate-based mineral mulches tested or spraying plants with phosphite improved plant growth and reduced root damage to a level comparable with phosphite (Supplementary Table S1). Plants from these treatments and the control pots with no soil additives were selected to analyze the soil microbiota (Table 1). Details of these treatments are Treatment 1, “No mulch”: pots with no additives; Treatment 2, “Organic mulches”: pots with well-composted chicken manure fertilizer, jarrah wood mulch for moisture retention, and mulch from an avocado orchard to simulate orchard conditions; Treatment 3, “Mineral mulch” organic mulches with the addition of a silicate-based mineral mulch (which also contained some calcium and trace elements such zinc, copper, boron) (https://mineralmulch.com/), and Treatment 4, “Phosphite” organic mulches and plants were sprayed with phosphite (Agri-Fos 600) (Nufarm Australia) to run off (Table 1).

**Table 1:** Experimental treatments.

**Treatment code**	**Chicken manure**	**Mulch**			**Chemical spray**
	50 g per pot applied monthly	Jarrah wood 75 g per pot at the time of transplantation	Avocado 75 g per pot at the time of transplantation	Mineral 100 g/pot at the time of transplantation	0.5% phosphite with 133 μl L^−1^ penetrant (BS-1000) applied to foliage 28 and 38 days after transplanting
No mulch	-	-	-	-	-
Organic mulches	+	+	+	-	-
Mineral mulch	+	+	+	+	-
Phosphite	+	+	+	-	+

At harvest, bulk soil was shaken gently from the roots, and then ~10 ml of the adhering rhizosphere soil was gently brushed into 15-ml freezing tubes (ThermoFisher scientific). Healthy white root tips (~1 ml) were randomly collected from each plant during harvesting and placed in a 1.5-ml Eppendorf tube. The rhizosphere soil samples and the root tips were immediately immersed in liquid nitrogen and stored at −20°C before DNA extraction.

### Analysis of the Soil and Plant Microbial Profile (Metabarcoding)

A total of 250 mg of rhizosphere soil and 50 mg of roots were used for the extraction of DNA using DNeasy^®^ PowerSoil^®^ Pro Kit (QAIGEN group) and DNeasy Plant Pro and Plant Kits (QAIGEN group), respectively following the manufacturer's instructions. One sample was analyzed from each rhizosphere and soil.

For taxonomic profiling, 16S primers (Klindworth et al., 2013) were used in combination for the amplification of the hypervariable V3 and V4 regions of the bacterial 16S rRNA gene following the procedures of 16 S RNA gene amplicon suggested for Illumina Miseq systems (Illumina documents 2019). For each sample, PCR amplicons were generated from three technical replicates using 25 μl mixtures containing 2.5 μl microbial genomic DNA, 1 μl of Illumina 16S forward and reverse primers each, 8 μl PCR grade water, and 12.5 μl of GoTraq^®^ green master mix per sample. This PCR was carried out on a thermal cycler and had a first denaturation cycle at 95°C for 3 min, 25 cycles of the second denaturation at 95°C for 30 s followed by primer annealing at 55°C for 30 s with further extension at 72°C for 30 s, at the end with a final step of heating at 72°C for 5 min. To reduce the variation in each PCR, amplicons of the technical replicates were pooled and cleaned up using AMPure XP beads (Beckman Coulter, USA) according to the manufacturer's instructions. After clean-up, PCR Illumina sequencing adapters were attached by an index PCR step using the Nextra XT Index kit (Illumina Inc., San Diego, CA USA). The mixture consisted of 5 μl DNA, 5 μl Nextra XP index Primers 1 and 2 each, 25 μl of 2 × KAPA HiFi Hotstart ReadyMix, and 10 μl PCR grade water. The PCR program was run on a thermal cycler using an initial denaturation step at 95°C for 3 min, followed by 8 cycles of the second denaturation at 95°C for 30 s, then primer annealing at 55°C for 30 s and further extension at 72°C for 30 s, and finally a heating cycle at 72°C for 5 min. Then the amplicons were again cleaned up following the same procedure described previously. The quantification of amplicon libraries was done using Qubit (Invitrogen, CA, USA), and all the samples were combined in equimolar amounts (4 mM each).

Library preparation was performed per the Illumina Guide for 16S Metagenomic Sequencing Library Preparation (Illumina). Indexed amplicon pools were sequenced on the Illumina MiSeq platform using 2 x 300 bp paired-end chemistry. Using the FASTA manifest protocol, de-multiplexed paired-end reads were imported into the Quantitative Insights into the Microbial Ecology platform (QIIME 2). Read trimming, primer removal, denoising, read merging, and amplicon sequence variant (ASV) clustering were performed using the DADA2 plugin pipeline. Taxonomy was assigned using the QIIME 2 feature classifier with the Greengenes v13.8 99% OTU 16s rRNA genes after removing chloroplast and mitochondrial operational taxonomic units (OTUs) from the dataset.

### Statistical Analysis

Statistical analysis was computed in R software (Version 4.1.1). Alpha diversity indices (Shannon indices) were calculated with the vegan package in R from the OTU table normalized by rarefaction to reduce the impact of sequencing depth on results. The alpha diversity indices were subjected to ANOVA to assess the difference between treatments and infection status, with statistical significance calculated from a permutation test. Linear-mixed effects model was used to calculate the species richness. Beta diversity (Bray-Curtis metric) indices were calculated through the vegan package. The effects of treatments and infection status on beta diversity indices were assessed with permutational multivariate ANOVA using distance matrices (*via* the “adonis” function through the vegan package). Non-metric multidimensional scaling (NMDS) based on UniFrac weighted and unweighted distances was performed using the vegan package, and plant root assessment factors were added to NMDS plots through the “envfit” function in the R vegan package. Data on total root weight, fine root weight, and root damage were from Farooq et al. (2022) (Supplementary Table S1).

A permutational multivariate ANOVA was used to compare the structure of bacterial communities of treatments with infection status using the vegan package unifrac weighted and unweighted distance matrix with 999 permutations. Significant differences in bacterial taxa relative abundance between treatments and infection status were calculated through the Kruskal–Wallis tests. The difference of relative abundance between infested and non-infested plants for each treatment and between treatments for each organism within a particular classification was compared by Mann-Whitney (Wilcoxon) tests. Tree-view of differences in the bacterial abundance was generated through the metacoder package in R. A cluster analysis to compare similarities of bacterial communities across the samples was undertaken based on the relative abundance data. Multivariate distances between samples were calculated using the vegan package in R and the cluster analysis with associated dendrogram and heatmap were generated from these distance data using the heatmap.2 function in the gplots package of R. Venn diagrams were generated to observe the shared and unique OTUs among the groups based on the prevalence of OTUs in sample groups, regardless of their relative abundance by using the VennDiagram package. A significance value of *p* < 0.05 was used where statistical testing was performed.

## Results

### Bacterial Diversity and Species Richness

**Figure 1:**
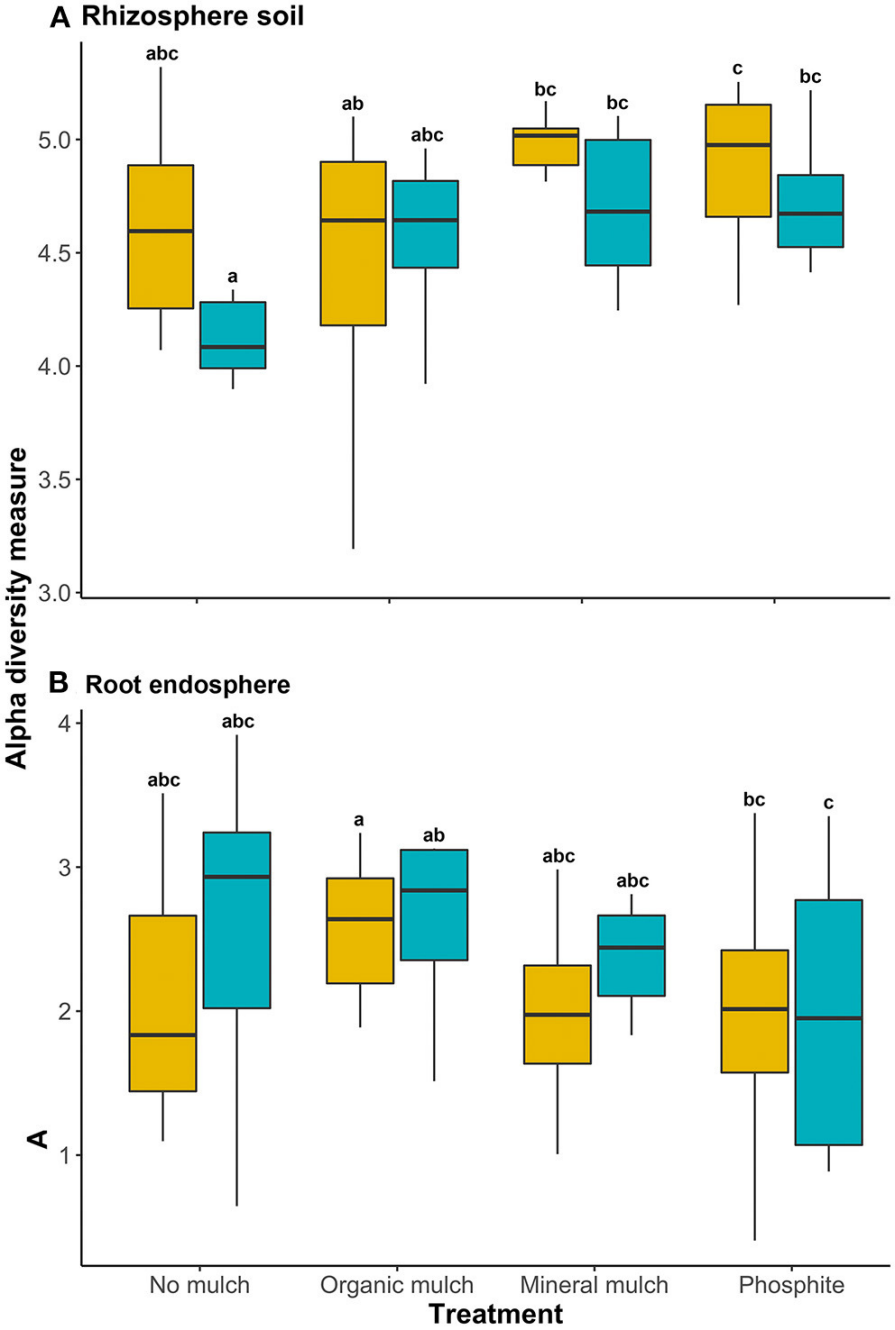
Alpha diversity of the microbial biome of **(A)** rhizosphere soil and **(B)** root endosphere of avocado plants given four soil treatments. Yellow boxplots show alpha diversity, measured by the Shannon diversity index for non-infested avocado, blue boxes represent plants infested with *Phytophthora cinnamomi*. Different lowercase letters indicate a significant (*P* < 0.05) difference.

In the rhizosphere, there were significant differences in the Shannon (alpha) diversity measure for bacterial communities between the four treatments (*p* = 0.03; Figure 1A), but there were no significant differences in alpha diversity indices for the root endospheres (Figure 1B). Adding mineral mulch or spraying plants with phosphite resulted in greater bacterial diversity in rhizospheres with greater consistency between pots than in the rhizosphere of plants from other treatments (Figure 1A).

**Figure 2:**
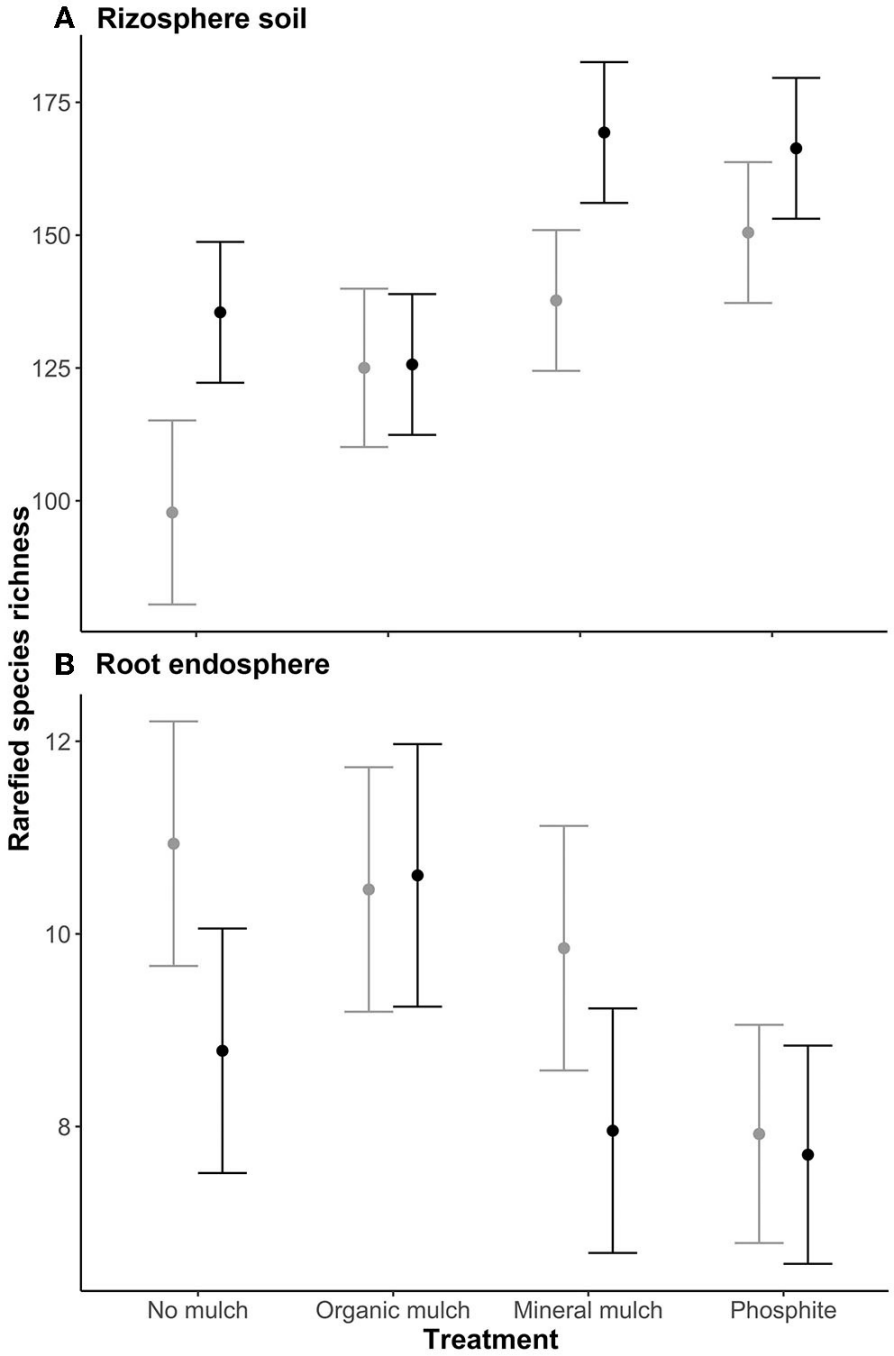
Mean rarefied species richness of the microbial biome of **(A)** rhizosphere soil, and **(B)** root endosphere of avocado plants given four soil treatments. Values are estimated marginal means ± standard error. Gray lines indicate values for non-infested plants, black lines plants infested with *P. cinnamomi*.

The linear mixed-effects model suggested that rarefied species richness in the rhizosphere of plants treated with mineral mulch or phosphite was significantly higher than those from organic mulch for non-infested plants, and significantly higher than those from no mulch or organic mulch for infested plants (Figure 2A). No significant differences in species richness in root endospheres were observed between treatments or between non-infested and infested (Figure 2B).

### Beta Diversity

**Figure 3:**
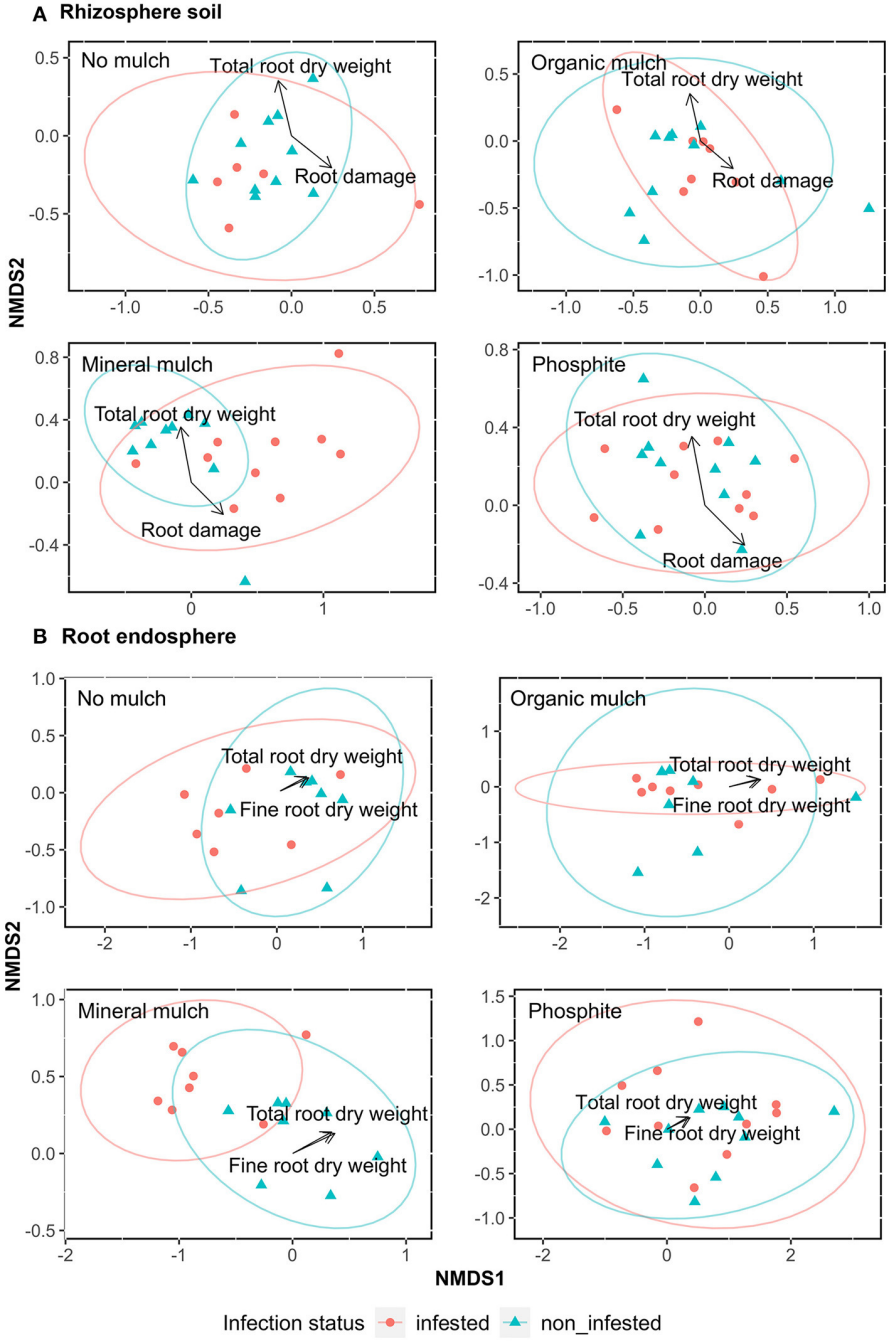
Association of bacterial communities with parameters of root health (total root dry weight, fine root dry weight and root damage) of avocado plants given four soil treatments. Non-metric multidimensional scaling (NMDS) showing the bacterial taxonomic clustering of microbes in **(A)** rhizosphere soil, and **(B)** root endosphere from the four treatments. Blue triangles indicate samples from non-infested and red dots treatments infested with *P. cinnamomi*. The analysis was based on the Bray-Curtis dissimilarity matrix using relative abundance data obtained from a Hellinger-transformation of the number of microbe reads. Data for root health was selected through Adonis PERMANOVA and were from Farooq et al. (2022) Supplementary Table S1.

Beta-diversity (Bray-Curtis metrics) indices of the microbial populations showed the greatest difference between non-infested and infested rhizosphere soil from plants with the mineral mulch treatment, with the first dimension (NMDS1) separating the infested from non-infested soil samples (Figure 3A). The NMDS plots showed low variability in bacterial communities between infested and non-infested plants in all other treatments and minimal separation of bacterial communities between treatments regardless of infection status.

There were significant differences among treatments regarding infection status in the beta analysis shown by Adonis analysis of the rhizosphere soil. For bacterial communities, the main effect of treatments and infection status and their interaction on beta-diversity were highly significant (Adonis) for rhizosphere soil. For bacteria, treatments, infection status and their interactions accounted for 7, 2, and 5% of the total variability, respectively. The interaction was primarily because the difference between infested and non-infested samples was greatest for mineral mulch.

### Explanatory Variables Significantly Related With NMDS Axis

To visualize the relationship between the variability in the community composition among the treatments with regard to infection status, the root damage fine root and total root dry weights were plotted in the NMDS ordination as fitted explanatory variables. The variation in community composition of rhizosphere soil was correlated significantly with root damage (*p* = 0.01, *R*^2^ = 0.02, F.mod = 1.61) and total root dry weight (*p* = 0.04, *R*^2^ = 0.01, F.mod = 1.39; Figure 3A). For root samples, the variation in community composition was significantly correlated to fine root dry weight (*p* = 0.01, *R*^2^ = 0.02, F.mod = 1.90) and total root dry weight (*p* = 0.03, *R*^2^ = 0.02, F.mod= 1.71; Figure 3B).

### Relative Abundance

**Figure 4:**
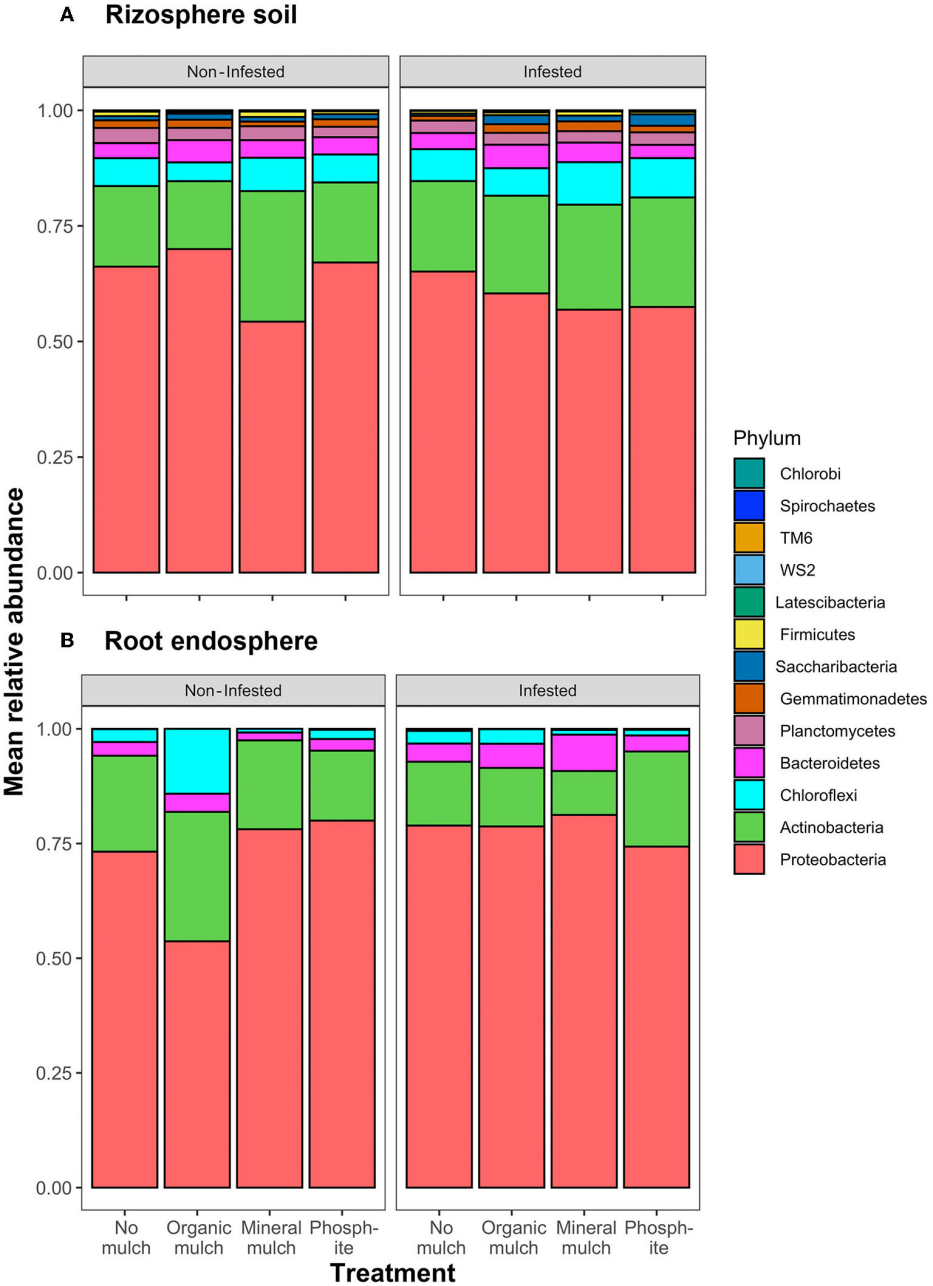
Relative abundance of phyla across the four treatments and comparing infested and non-infested plants in **(A)** rhizosphere soil and **(B)** root endosphere.

In rhizosphere soil, significant differences in relative abundance were observed for the Actinobacteria and Proteobacteria across the four treatments for non-infested plants, with the relative abundance of Proteobacteria being lower, and Actinobacteria higher than in the other treatments (Figure 4A, Table 2). Comparing relative abundance in infested and non-infested rhizospheres from the same soil treatment indicated little difference in the treatment without mulch. In the mineral mulch and phosphite treatments, Actinobacteria increased (28%) in abundance, while they decreased (15%) in the organic mulch treatment. There was also a significant increase in Spirochaetes in infested plants from treatments with organic mulch, mineral mulch, or phosphite compared with no mulch (Figure 4A, Supplementary Figure S1A, Table 2). In root endospheres, there were significantly fewer phyla present. In the non-infested root endosphere from the organic mulch treatment, the proportion of Chloroflexi was high (7%), but the increase was not statistically significant, whereas in infested root endosphere, the relative abundance of Bacteroidetes was significantly higher in the mineral mulch treatment (7.9%) than in other treatments (ranging from 2 to 4%). A major difference between non-infested and infested root endosphere was seen between those from the mineral mulch treatment in which non-infested endophytic roots had fewer Actinobacteria (36%) and Bacteroidetes (93%) than the infested root endosphere (Figure 4B, Supplementary Figure S1B, Table 2).

**Table 2:** Comparison of relative abundance of bacterial communities between treatments within each infested/non-infested group tested by Kruskal-Wallis test.

**Phylum**	**Infection status**	**Rhizosphere soil**	**Root endosphere**
Actinobacteria	non-infested	0.0003	
Proteobacteria	non-infested	0.0001	
Spirochaetes	infested	0.0120	
Bacteroidetes	non-infested		0.0080
Chloroflexi	non-infested		0.0018

*Only p < 0.05 for significant differences between treatments are displayed*.

Analysis of relative abundance of the bacteria at the genus level (Supplementary Data Sheets S1A,B) illustrates the significant difference in the bacterial profiles resulting from mineral mulch or phosphite treatment. In non-infested rhizospheres, species of 11 genera significantly increased in relative abundance in response to both mineral mulch or phosphite treatments, 2 in phosphite but not mineral mulch, and 17 in mineral mulch but not phosphite. When the changes after infestation are examined further, there is a large difference between the groups that increase in relative abundance: in mineral mulch, three Actinobacteria, two Chloroflexi, two Gamma Proteobacteria, and six Proteobacteria increased relative to the levels in non-infested pots; whereas in phosphite-treated soils, only one Actinobacteria and one Proteobacteria genus increased in relative abundance. No distinctive clustering of bacterial communities was observed in heatmaps of rhizosphere soil samples for relative abundance among treatments and infection status groups (Supplementary Figure S4A). A more “continuous” variation in the biological samples of soils was found as indicated by the more uniform color of the heatmap and the structure of the accompanying dendrogram, as expressed by their relative abundance. Nonetheless, a small level of clustering of bacterial communities was present in mineral mulch compared with phosphite (Supplementary Figure S4A).

In contrast, there was a very distinctive clustering of bacterial communities in the heat maps of the endophytic root bacteria, indicating a similarity of groups of samples as expressed by their relative abundances. However, despite this, clustering was not related to treatments or infection status in a pairwise comparison of treatments (Supplementary Figure S4B).

### Total Abundance

In the rhizospheres of plants in non-infested soil, the addition of organic mulch did not increase the bacterial abundance; however, the addition of mineral mulch or phosphite treatment did cause a rise, with a marked increase in the number of Actinobacteria in the mineral mulch treatment and Proteobacteria in both treatments (Supplementary Figures S1A, S2). Bacteria were less abundant in all treatments in infested soils, but there was an abundant increase with the application of organic mulch, and there were additional rises with mineral mulch and phosphite treatments.

In the root endosphere, bacterial abundance was eight times lower than in the rhizosphere, and fewer phyla were represented. In the roots from non-infested soil, organic mulch resulted in a doubling of the endophytic bacterial population with a marked increase in the abundance of Actinobacteria and Chloroflexi, but the other treatments were similar to the no mulch treatment. There were more bacteria present in the root endosphere from infested soils with the mineral mulch treatment having high levels of Proteobacteria and Bacteroidetes (Supplementary Figures S1B, S3).

### Comparison of the OTUs Present in Different Treatments

**Figure 5:**
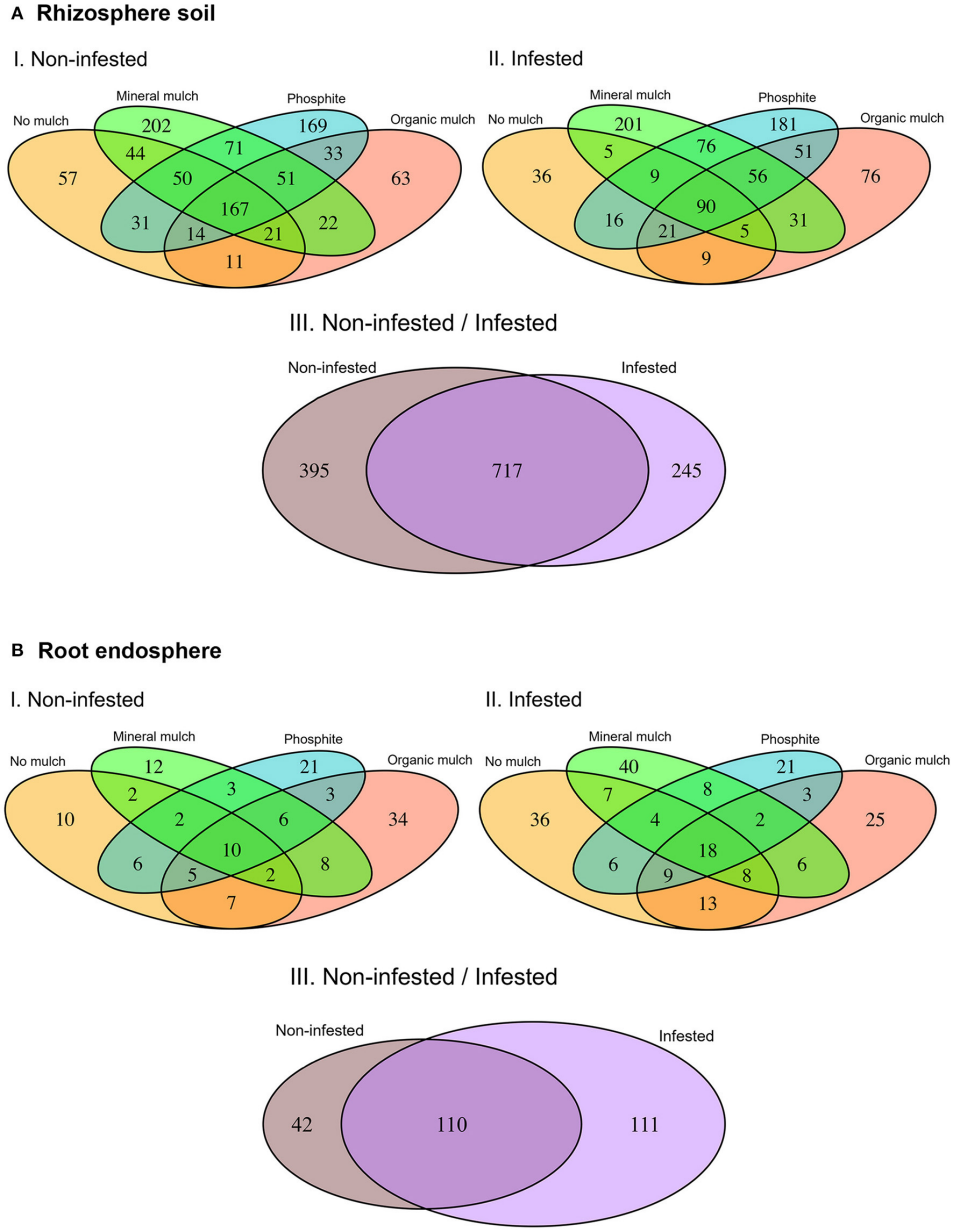
Venn diagrams representing the operational taxonomic units (OTUs) in common and different in the microbiome of **(A)** rhizosphere soil and **(B)** root endosphere among treatments.

Differences in the composition of the bacterial microbiome and identification of the OTUs unique to a soil treatment or common across treatments are presented in the Venn diagrams (Figure 5A I, II, III). In non-infested rhizosphere soil, total OTUs were the least in treatments with no mulch or avocado mulch, and highest in the treatments with mineral mulch (65% of the total OTUs) or phosphite (60% of the total OTUs), which also had much higher numbers of unique OTUs (Figure 5A I). In infested soil, there was a reduction (10%) in the total number of OTUs in all treatments, but again, the highest total number and number of unique OTUs were in treatments with mineral mulch (54% of total OTUs) or phosphite (57% of total OTUs) (Figure 5A II). The number of OTUs common to all treatments in non-infested soil was 167 (17% of total OTUs), dropping to 90 (10% of total OTUs) in infested soils. The eight phyla that occurred in all treatments were Actinobacteria, Bacteroidetes, Chloroflexi, Firmicutes, Gemmatimonadetes, Planctomycetes, Proteobacteria, and TM7 (Saccharibacteria). When the data from treatments were bulked, the number of OTUs common to infested and non-infested treatments were higher than the number unique to either treatment (Figure 5A III).

Overall, endophytic roots had many fewer OTUs than the rhizosphere. Some of the main phyla found in rhizosphere soil such as TM6, Latescibacteria, Spirochaetes Planctomycetes, Firmicutes and Chlorobi were absent from roots samples. No mulch and mineral mulch treatments had the highest numbers, and the proportion of OTUs common to non-infested and infested treatments was higher than in the rhizosphere (Figure 5B I, II, III). The four phyla that occurred in all treatments were Actinobacteria, Bacteroidetes, Chloroflexi, and Proteobacteria. The number of OTUs unique to root endospheres was 8% of the total OTUs found in soil and root combined.

## Discussion

### The Effect of Additives on Bacterial Diversity and Abundance

The abundance and diversity of the soil microbiome were greatest in treatments in which *Phytophthora* root damage was suppressed, that is, soils from plants supplied with mineral mulch or in which plants were sprayed with phosphite. The NMDS plots showed the strongest link between root health and the variability of the rhizosphere microbes was in the treatment with mineral mulch and this microbiome showed more difference between the non-infested and infested pots than any other treatment. The total number of OTUs was highest in treatment with mineral mulch or phosphite, although infection *with P. cinnamomi* reduced the total number of OTUs detected, and in both non-infested and infested treatments the highest numbers of OTUs were in the mineral mulch and phosphite treatments. Of note, a strong clustering of bacterial communities was seen in the root samples but not in the rhizosphere, as shown from the heatmaps. This is in contrast to most of the other findings in this study where differences in diversity, species richness, and abundances were found in root samples but not in the rhizosphere.

Several studies have reported a decrease in microbial abundance and diversity in the root endosphere or the rhizosphere with infection. Yang and Ancona (2021) found a decrease in abundance and diversity of microbial communities in the root endosphere after infection with *P. nicotianae* in *Citrus*. Liu et al. (2020) observed a reduction in microbial diversity in the root endosphere after infection with *P. nicotianae* in *Nicotiana tabacum*, but the opposite occurred in the soil. The decrease in bacterial composition in soil was also observed by Byers et al. (2020) in kauri forest soils in which *P. agathidicida* was present, and Wei et al. (2021) found that diseased cotton plants infested with *Verticillium* wilt had a higher total microbial biomass than healthy plants but less bacterial diversity.

### Changes in the Microbiome Due to the Additives

The total number of OTUs detected in the various treatments indicated that the diversity was not markedly increased by the addition of organic mulch, but was significantly increased when mineral mulch or phosphite treatments were added. Mean relative abundance of different phyla in the rhizosphere was not markedly affected by the addition of organic mulch or spraying the plants with phosphite. However, mineral mulch caused a significant drop in the proportion of Protobacteria and an increase in Actinobacteria in non-infested treatments. When *P. cinnamomi* was present, the rhizospheres from soils with mineral mulch or phosphite, both had less Proteobacteria but more Actinobacteria and Chloroflexi than the treatments with no mulch or organic mulch treatments.

The abundance and diversity of the bacterial microbiomes in the root endospheres were much lower than in the rhizosphere in all treatments. There was a marked increase in Chloroflexi in non-infested pots with organic mulch, but this was not seen in the corresponding infested treatment. In the infested roots, the proportions of endophytic Actinobacteria and Bacteroidetes were different in the two treatments most suppressive of *Phytophthora*, namely the mineral mulch and phosphite treatments.

Actinobacteria, a phylum whose relative abundance increases in effective treatments, such as mineral mulch and phosphite, includes taxa such as *Streptomyces* known to produce antibiotic compounds effective against *Phytophthora* (Koberl et al., 2013). Isolates have also been shown to control *P. drechsleri* damping-off in cucumber (Sadeghi et al., 2017). However, *Streptomyces* species did not increase with the treatments applied in the current experiments.

Other genera that include species known to suppress fungal pathogens and *Phytophthora* by producing a wide range of antibiotic or volatile compounds, siderophores, and growth-promoting compounds are *Pseudomonas* and *Burkholderia* in the Proteobacteria (Inderbitzin et al., 2018; Youseif, 2018). In the current study, the proportion of this group was reduced by the treatments most effective against *Phytophthora*. In many other studies, *Pseudomonas* spp. have been shown to be effective for the control of *Phytophthora* (Broadbent et al., 1971; Broadbent and Baker, 1974; Duvenhage et al., 1991; You et al., 1996; Yang et al., 2001; Hunziker et al., 2015; Martins et al., 2021). In the current study, *Pseudomonas* spp. were not more abundant in the effective treatments (mineral mulch and phosphite) than in organic mulch alone before infection; however after infection, *Pseudomonas* spp. were more abundant in mineral mulch treatment. *Bukholdaria* was more abundant in phosphite but not mineral mulch. Thus, the changes in the microbial profiles following treatment with mineral mulch or phosphite were very different.

### Impact of *P. cinnamomi* on the Bacterial Microbiome

Infestation of the soil with *P. cinnamomi* resulted in a decrease in OTUs in all treatments. However, the decrease was accompanied by different alterations in the relative abundance of the phyla in the various treatments. Many more genera changed in abundance at the genus level in response to infection in the mineral mulch treatment compared with phosphite. There was no significant change in the abundance of *Streptomyces, Pseudomonas*, or *Burkholdaria* after infestation in the treatments with no mulch or phosphite.

It was hypothesized that as avocado root damage from *Phytophthora* is reduced to a similar extent by silicate-based mineral mulch or spraying plants with phosphite, then both treatments would induce similar changes in the soil microbiome. However, the relative abundance of the various phyla in these two treatments was very different. In non-infested rhizospheres, the proportion of OTUs in common between these two treatments was similar to that of ineffective treatments. The dissimilarity of genera increase in abundance after adding mineral mulch or phosphite, and the changes following infection indicate that each treatment affects different bacterial taxa. Taxa shown to be important for disease control in other studies did not appear in this study and it appears there are many genera worthy of further study concerning whether they protect plants from *Phytophthora* root rot.

Many studies have shown that the addition of organic matter improves the physical and chemical properties of soil (Duffy et al., 1997; Tenuta and Lazarovits, 2002), change bacterial diversity, composition, and overall activity (Van and Van Ginkel, 2001; Bulluck and Ristaino, 2002; Cohen et al., 2005; Perez-Piqueres et al., 2006), as well as increasing the disease resistance of plants (Bonanomi et al., 2010; Aviles et al., 2011). However, in the current experiment, the addition of organic mulch (the chicken manure, jarrah mulch, and avocado mulch) increased the total abundance and changed the relative abundance of bacterial phyla present in the rhizosphere but did not result in significantly less damage from *P. cinnamomi*. Although the potting medium was designed to replicate field conditions as far as possible in the field, greater fluctuations in temperature and moisture may alter the responses of both the microbiome and the plants.

### Possible Modes of Action of the Suppressive Treatment–Mineral Mulch and Phosphite

The mineral mulch contained silicon and calcium, compounds known to improve bacterial diversity and richness significantly and in particular, to increase the relative abundance of Proteobacteria and Actinobacteria (Samaddar et al., 2019; Chen et al., 2021). An increase in Actinobacteria was also observed in the current study, but there was a decrease in the relative abundance of Proteobacteria. Silicon may also improve the production of plant metabolites that are associated with plant defense mechanisms against pathogens (Cherif et al., 1994; Rahman et al., 2015). When plant defensive mechanisms are activated, root exudates change, which impacts the soil microbiome (Ansari, 2018; Hu et al., 2018). Silicon may also decrease the disease severity by creating a physical barrier between the cell wall and cuticle (Song et al., 2021). Silicon is an environmentally friendly treatment and can be used in organic farming (Artyszak, 2018).

Phosphite, like silica, activates plant defense mechanisms (Ramezani et al., 2018; Mohammadi et al., 2020) and also has a direct effect on the pathogen. The changes in microbiome seen after phosphite spraying on the leaves are likely to be mediated through changes in root exudates (Eshraghi et al., 2014; Achary et al., 2017; Gill et al., 2018). The addition of phosphite increased the abundance of soil bacteria, but the relative abundance of all the phyla was very similar across the treatments. Su et al. (2021a) examined the effects of phosphite on the microbiome of tomatoes and found it enhanced the abundance of Proteobacteria and Actinobacteria.

### Endophytic Bacteria in Roots Compared to the Rhizosphere

The rhizosphere soil had more taxa and thus greater bacterial diversity and richness than the root endosphere (as shown by Venn diagrams and alpha diversity). In a previous study by Cordero et al. (2020), infection with *P. cinnamomi* resulted in an increase in the number of endophytic microbes in the roots and in the number of unique OTUs in many crop species. However, in the current study, although the number of microbes was increased after infection with *P. cinnamomi*, the total number of OTUs in the roots endosphere remained much lower than the rhizosphere soil.

As the roots were not surface-sterilized before analysis, the bacteria detected included some from the rhizosphere as well as endophytic organisms. Of the total root OTUs 30–50% were not detected from the comparable rhizosphere, suggesting that up to half of the microbes have a preference for, or are only found as endophytes in roots. These include the Actinobacteria (e.g., *Williamsia serinedens, Streptomyces reticuliscabie*, and *Goprdonia* sp.), Bacteroidetes (e.g., *Flavobacterium succinicans, Mucilaginibacter* sp., and *Sphingobacteriia* sp.), and Proteobacteria (e.g., *Sphiingomonas wittichii, Rhizobium giardinii*, and *Asticcacaulis* sp.). Several phyla that occur in the rhizosphere (Chlorobi, Firmicutes, TM6, Planctomycetes, Latescibacteria, and Spirochaetes) were absent from the roots. Yang and Ancona (2021) examined the endophytic bacteria in *Citrus* roots from healthy trees and those infested with *P. nicotianae*. They also recorded a decrease in the bacterial abundance and diversity resulting from infection. However, in their case, the relative abundance of Bacteroidetes increased and that of Proteobacteria decreased, whereas the opposite was the case in the avocado roots studied here. In roots of species in each of the unrelated plant genera *Persea, Citrus*, and *Arabis*, the most abundant root bacteria are from the phyla Proteobacteria, Actinobacteria, and Bacteroidetes but they differ in that in *Arabis*, Firmicutes, and in *Citrus*, Acidobacteria are also common phyla (Dombrowski et al., 2017; Zhang et al., 2017).

## Conclusion

The observed similarity in the reduction of *Phytophthora* root damage through the application of mineral mulch or phosphite was not linked to parallel changes in the rhizosphere or root microbiome. It is not possible to determine the relative importance of changes to the soil microbiome and changes to plant metabolism induced by the application of silica or phosphite. Changes to the soil microbiome are important, and they are different after the application of silica or spraying with phosphite. Analysis of the whole microbiome suggested that a wide range of bacteria may be involved in suppressing *Phytophthora* root rot, but the major genera are likely to come from the phyla Actinobacteria, Firmicutes, and Proteobacteria. Further insight into the crucial groups involved will be obtained from RNA sequencing.

## Data Availability Statement

The data sets related to this study can be found in online repositories. The names of the repository/repositories and accession number(s) can be found below: https://data.mendeley.com/drafts/h7jxb8frm2, 10.17632/h7jxb8frm2.1.

## Author Contributions

QF, JM, GH, and TB designed the experiments. QF carried out the experiments. Data were analyzed by QF and PT. All authors contributed equally to the manuscript and agreed to the submitted version.

## Funding

This research was supported by Murdoch University Scholarship and HIA (Horticulture Innovation Australia) project AV10067.

## Conflict of Interest

The authors declare that the research was conducted in the absence of any commercial or financial relationships that could be construed as a potential conflict of interest.

## Publisher's Note

All claims expressed in this article are solely those of the authors and do not necessarily represent those of their affiliated organizations, or those of the publisher, the editors and the reviewers. Any product that may be evaluated in this article, or claim that may be made by its manufacturer, is not guaranteed or endorsed by the publisher.

## Supplementary Material

Supplementary Figure S1 Total abundance of phyla across the four treatments and comparing infested and non-infested plants in a) rhizosphere soil and b) root endosphere.

Supplementary Figure S2 Difference between proportions of reads observed across the four treatments and comparing infested and non-infested plants in rhizosphere soil. The gray taxa on the lower left side represents a key for unlabelled trees. Each mini tree represents a comparison across the treatments in the rows and columns. Taxa colored in green are more abundant in treatments of the row and taxa in tan color are more abundant in treatments in columns. For figure a) and b) the color of each taxon represents the log-2 ratio of median proportion of reads observed in each treatment. Taxa colored in blue are abundant in the treatment mineral mulch whereas taxa colored in tan are more abundant in no mulch. Taxa in gray showed no difference across treatments.

Supplementary Figure S3 Difference between proportions of reads observed across the four treatments and comparing infested and non-infested plants in root endosphere. The gray taxa on the lower left side represents a key for unlabelled trees. Each mini tree represents a comparison across the treatments in the rows and columns. Taxa colored in green are more abundant in treatments of the row and taxa in tan color are more abundant in treatments in columns. For figure a) and b) the color of each taxon represents the log-2 ratio of median proportion of reads observed in each treatment. Taxa colored in blue are abundant in the treatment mineral mulch whereas taxa colored in tan are more abundant in no mulch. Taxa in gray showed no difference across treatments.

Supplementary Figure S4 Cluster analysis of **(A)** rhizosphere soil and **(B)** root endosphere bacterial samples across the four treatments. Samples with +Pc indicates those infested with *Phytophthora cinnamomi* and -Pc indicates non-infested.

Supplementary Table S1 The effect of four soil treatments on shoot dry weight, total root dry weight, fine root dry weight and root damage of non-infested avocado plants or those infested with *Phytophthora cinnamomi*. Significant differences between means for each growth parameter are indicated by different superscript letters. For each parameter, means for non-infested and infested plants in the same treatment that are significantly (*P* < 0.05) different are shown in bold. Root damage scores are 1 (least damaged) to 5 (most damaged). Data from Farooq et al. (2022).

Supplementary Data Sheet S1 **(A)** A comparison of the bacterial communities present (*OTU*s) and relative abundance within a particular classification (up to genus level) in non-infested and infested rhizospheres (for each of the four treatments) for which the abundance changed significantly following infection. Significance of differences were assessed by a Mann-Whitney (Wilcoxon) test with significant differences having *P* < 0.05. “-” indicates that the relative abundance at infested rhizosphere had a significantly lower relative abundance than in non-infested rhizosphere, when collapsed to the particular classification level, while a “+” indicates a significantly higher abundance at non-infested rhizosphere, respectively. The columns “n-” ad “n+” refer to the number of OTUs within a genus where an infested rhizosphere had a significantly lower (-) or higher (+) relative abundance, respectively. **(B)** A comparison of the relative abundance of bacterial communities within a particular classification present in the rhizosphere of non-infested pots with only organic mulch and those with, in addition, mineral mulch or in which plants were treated with phosphite. The columns “n-” and “n+” refer to the number of OTUs within a genus where organic mulch treatment had a significantly lower (-) or higher (+) relative abundance, than the mineral mulch or phosphite treatments, respectively.
