# Supplementary figures and images for: Changes to the Bacterial Microbiome in the Rhizosphere and Root Endosphere of Persea americana (Avocado) Treated With Organic Mulch and a Silicate-Based Mulch or Phosphite, and Infested With Phytophthora cinnamomi
Source: Front Microbiol. 2022 Apr 28;13:870900. doi: 10.3389/fmicb.2022.870900 (PMC9097018; doi:10.3389/fmicb.2022.870900)

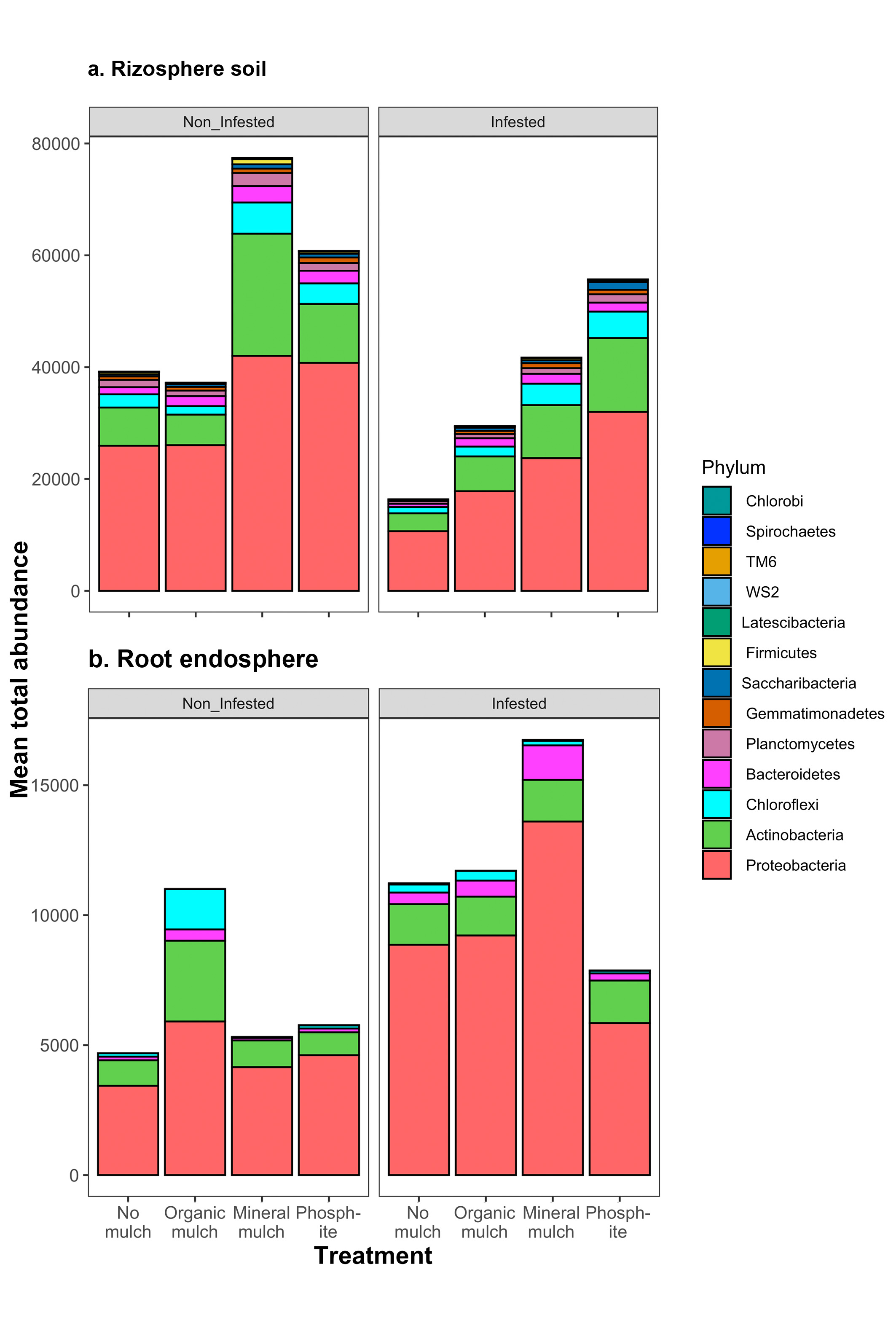

Supplement: Supplementary Figure S1 — Total abundance of phyla across the four treatments and comparing infested and non-infested plants in a) rhizosphere soil and b) root endosphere. [file Image_1.JPEG]

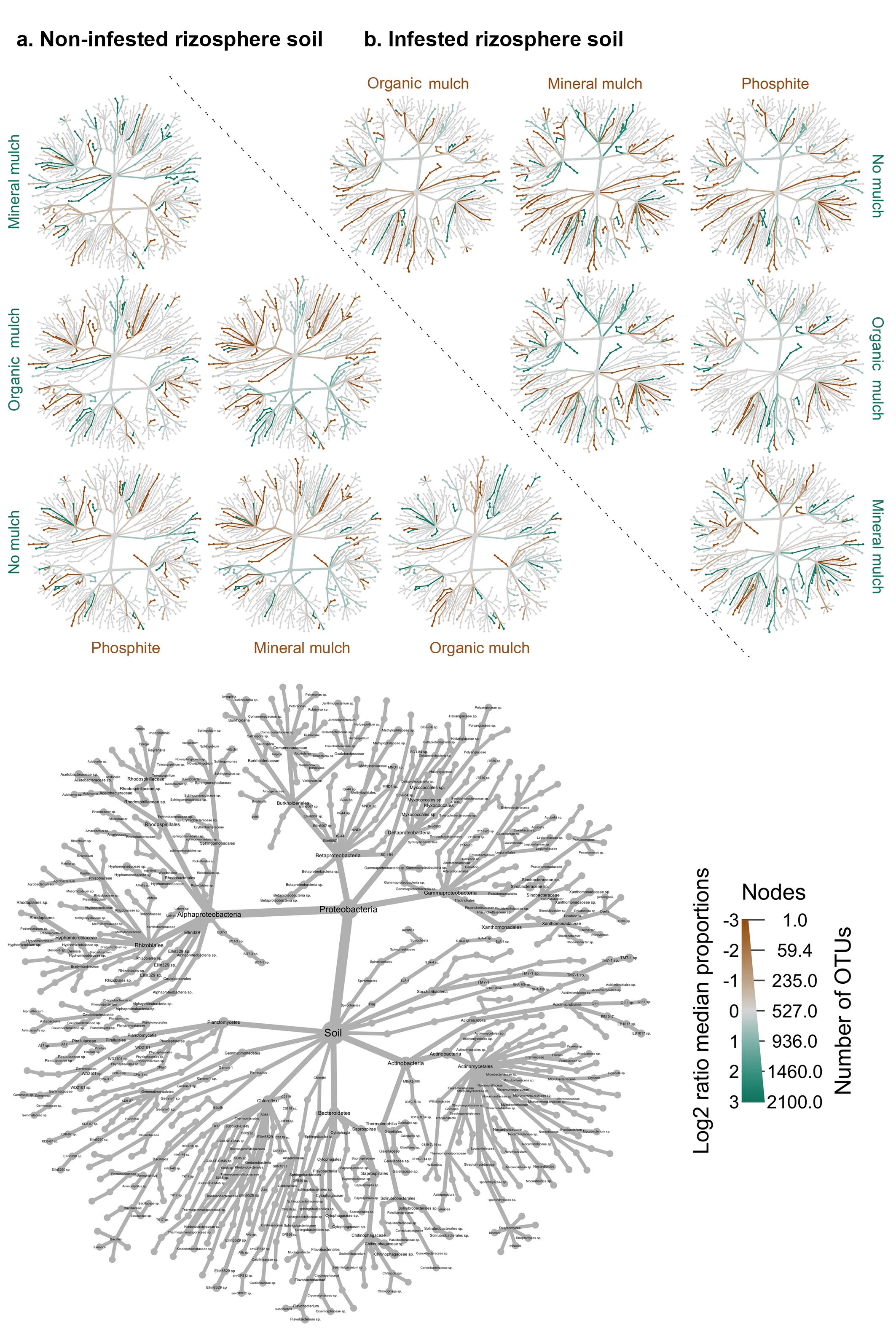

Supplement: Supplementary Figure S2 — Difference between proportions of reads observed across the four treatments and comparing infested and non-infested plants in rhizosphere soil. The gray taxa on the lower left side represents a key for unlabelled trees. Each mini tree represents a comparison across the treatments in the rows and columns. Taxa colored in green are more abundant in treatments of the row and taxa in tan color are more abundant in treatments in columns. For figure a) and b) the color of each taxon represents the log-2 ratio of median proportion of reads observed in each treatment. Taxa colored in blue are abundant in the treatment mineral mulch whereas taxa colored in tan are more abundant in no mulch. Taxa in gray showed no difference across treatments. [file Image_2.JPEG]

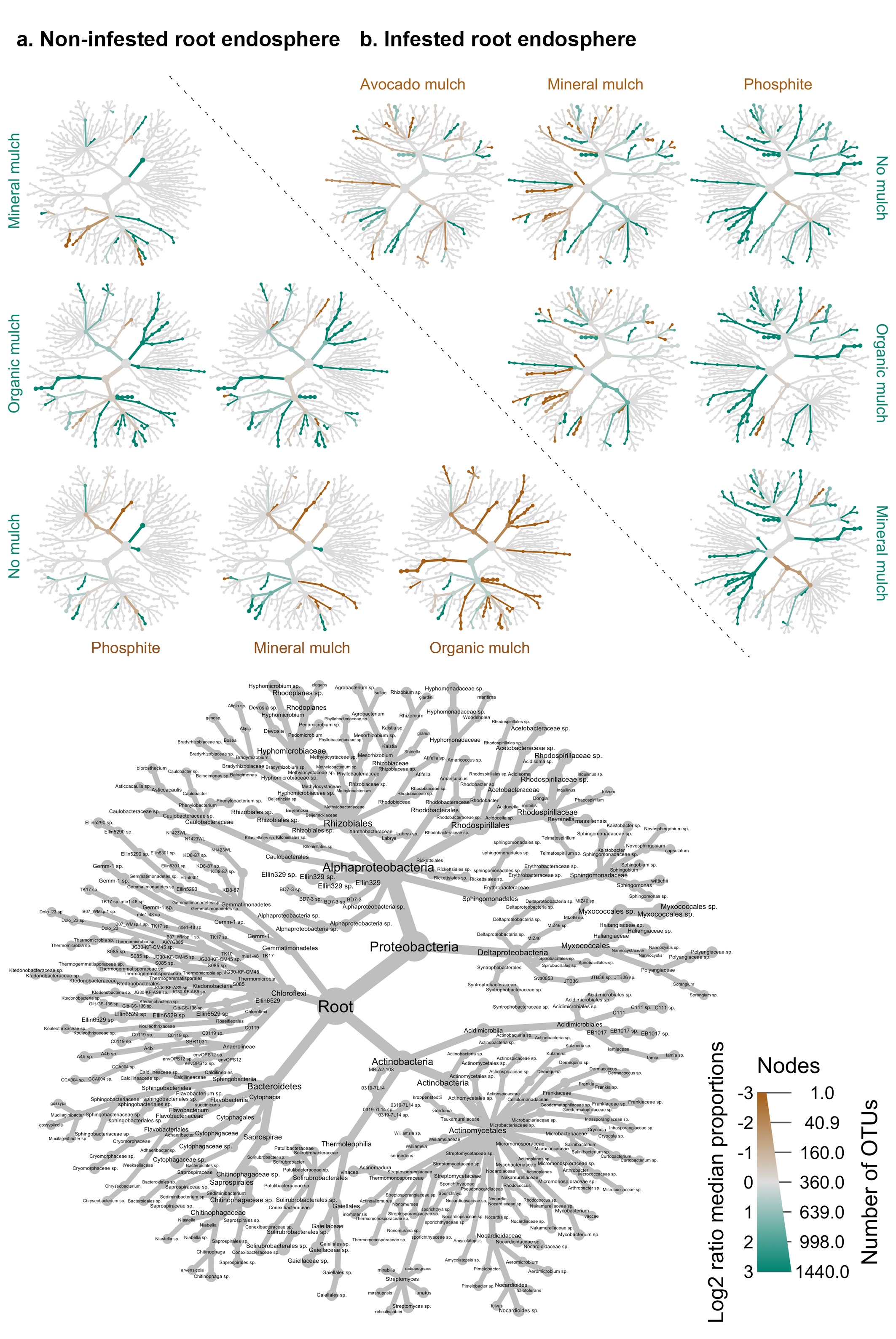

Supplement: Supplementary Figure S3 — Difference between proportions of reads observed across the four treatments and comparing infested and non-infested plants in root endosphere. The gray taxa on the lower left side represents a key for unlabelled trees. Each mini tree represents a comparison across the treatments in the rows and columns. Taxa colored in green are more abundant in treatments of the row and taxa in tan color are more abundant in treatments in columns. For figure a) and b) the color of each taxon represents the log-2 ratio of median proportion of reads observed in each treatment. Taxa colored in blue are abundant in the treatment mineral mulch whereas taxa colored in tan are more abundant in no mulch. Taxa in gray showed no difference across treatments. [file Image_3.JPEG]

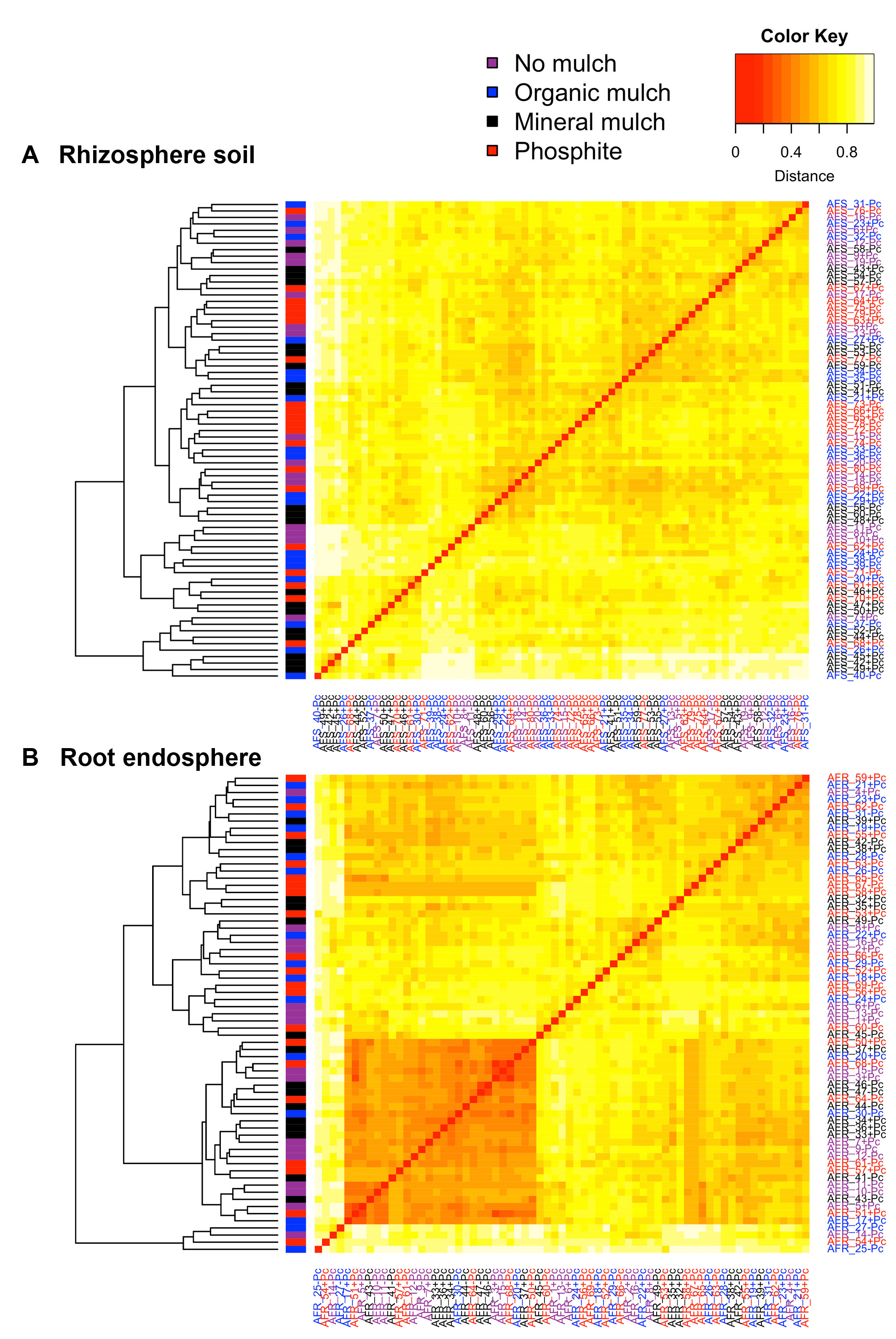

Supplement: Supplementary Figure S4 — Cluster analysis of (A) rhizosphere soil and (B) root endosphere bacterial samples across the four treatments. Samples with +Pc indicates those infested with Phytophthora cinnamomi and -Pc indicates non-infested. [file Image_4.jpg]
