# Supplementary material for: Changes to the Bacterial Microbiome in the Rhizosphere and Root Endosphere of Persea americana (Avocado) Treated With Organic Mulch and a Silicate-Based Mulch or Phosphite, and Infested With Phytophthora cinnamomi
Source: Front Microbiol. 2022 Apr 28;13:870900. doi: 10.3389/fmicb.2022.870900 (PMC9097018; doi:10.3389/fmicb.2022.870900)
Supplement: Supplementary Table S1 — The effect of four soil treatments on shoot dry weight, total root dry weight, fine root dry weight and root damage of non-infested avocado plants or those infested with Phytophthora cinnamomi. Significant differences between means for each growth parameter are indicated by different superscript letters. For each parameter, means for non-infested and infested plants in the same treatment that are significantly (P < 0.05) different are shown in bold. Root damage scores are 1 (least damaged) to 5 (most damaged). Data from Farooq et al. (2022). [file Table_1.DOCX]

Supp Table 1 The effect of four soil treatments on shoot dry weight, total root dry weight, fine root dry weight and root damage of non-infested avocado plants or those infested with *Phytophthora* *cinnamomi*. Significant differences between means for each growth parameter are indicated by different lower-case letters. For each parameter, means for non-infested and infested plants in the same treatment that are significantly different (P< 0.05) are shown in bold. Root damage ranks are 1 least damage, 5 most damage. Data from (Farooq et al 2022)

Duncan

| **T** | **Mean shoot dry weight (**±**SE) (g)** | | **Mean total root dry weight (**±**SE) (g)** | | **Mean fine root DW**  **(**±**SE) (g)** | | **Mean root damage**  **(**±**SE) rank 1-5** | |
| --- | --- | --- | --- | --- | --- | --- | --- | --- |
|  | **Non-infested** | **Infested** | **Non-infested** | **Infested** | **Non infested** | **infested** | **Non-infested** | **Infested** |
| No mulch | 49.5**±**4.8^ab^ | 39.7**±**4.22^b^ | **15.9±1.01^b^** | **10.6±0.51^c^** | 3.9±0.58^bc^ | 2.872±0.30^c^ | **1.5±0.22** | **4.0±0.33** |
| Organic mulch | 49.7**±**5.01^ab^ | 40.5**±**4.17^b^ | **15.9±1.48^b^** | **10.7±1.18^c^** | **4.6**±**0.58^b^** | **2.8**±**0.29^c^** | **1.4±0.16** | **3.8±0.38** |
| Mineral mulch | 63.7±5.68^a^ | 50.9±4.94^ab^ | **22.5±1.27^a^** | **17.6±1.27^b^** | **7.4±0.53^a^** | **4.8±0.66^b^** | **1.0±0.00** | **2.9±0.38** |
| Phosphite spray | 61.5±3.71^a^ | 49.9±3.24^ab^ | **22.0±1.08^a^** | **16.7±0.73^b^** | **7.6±0.60^a^** | **4.2±0.26^bc^** | **1.1±0.10** | **3.1±0.37** |
